# Analysis of Facilitators and Barriers to the Delivery of Routine Care during the COVID-19 Global Pandemic: A Systematic Review

**DOI:** 10.3390/healthcare9050528

**Published:** 2021-05-01

**Authors:** Cristian Lieneck, Brooke Herzog, Raven Krips

**Affiliations:** School of Health Administration, Texas State University, San Marcos, TX 78666, USA; bzh4@txstate.edu (B.H.); rjk54@txstate.edu (R.K.)

**Keywords:** routine care, COVID-19, coronavirus, global pandemic

## Abstract

The delivery of routine health care during the COVID-19 global pandemic continues to be challenged as public health guidelines and other local/regional/state and other policies are enforced to help prevent the spread of the virus. The objective of this systematic review is to identify the facilitators and barriers affecting the delivery of routine health care services during the pandemic to provide a framework for future research. In total, 32 articles were identified for common themes surrounding facilitators of routine care during COVID-19. Identified constructed in the literature include enhanced education initiatives for parents/patients regarding routine vaccinations, an importance of routine vaccinations as compared to the risk of COVID-19 infection, an enhanced use of telehealth resources (including diagnostic imagery) and identified patient throughput/PPE initiatives. Reviewers identified the following barriers to the delivery of routine care: conservation of medical providers and PPE for non-routine (acute) care delivery needs, specific routine care services incongruent the telehealth care delivery methods, and job-loss/food insecurity. Review results can assist healthcare organizations with process-related challenges related to current and/or future delivery of routine care and support future research initiatives as the global pandemic continues.

## 1. Introduction

### 1.1. Rationale

The COVID-19 global pandemic continues to challenge healthcare industries across the globe, with adaptations to the delivery of care to enforce public health initiatives ongoing. Physical distancing remains a vital task in preventing the spread of COVID-19, while other related variables stress the ability for healthcare systems to deliver routine care services [1,2,3]. With many healthcare organizations focusing solely on the delivery of urgent (acute) care to control the prevalence of the disease, routine health care services are often reprioritized by multiple industry stakeholders, to include the patients themselves [4,5]. While supporting physical distancing and other initiatives, the postponement of routine care may continue to delay the inevitable: an eventual influx of compounded chronic condition aliments that require urgent medical attention once the pandemic subsides.

Barriers influencing the delivery of routine care continue to exist, and many healthcare organizations continue to focus on adapted strategies and patient throughput models that enable the delivery of routine care during the pandemic. To continue to enforce public health initiatives, routine care services are often adjusted and/or adapted for pandemic precautions to be conducted in a safe manner while maintaining physical distancing and related preventive measures [6]. These best practices are often able to be adopted between healthcare organization types, and possibly between health care specialties and/or varying services lines.

### 1.2. Objectives

The purpose of this review is to identify and classify facilitators and barriers related to the delivery of routine care during the COVID-19 global pandemic. Furthermore, this information may be beneficial for healthcare organizations to assess their current levels of routine care being delivered and the potential for enabling medical personnel in establishing additional routine care services. As the number of individuals receiving the COVID-19 vaccination increases daily, organizations should be working to address any potential backlogs of patients who have delayed routine care services, yet also continuing to abide by local, regional, and/or national public health requirements.

## 2. Materials and Methods

### 2.1. Eligibility Criteria

This reviewed was guided by the Preferred Reporting Items for Systematic Reviews and Meta-Analysis (PRISMA) model. Articles were initially considered eligible in the study if they focused upon the delivery of routine care during the COVID-19 global pandemic. Articles had to be published in quality (peer-reviewed) journals and with publication dates between 1 January 2020 through 31 October 2020. This narrow publication window to permit eligibility into the study sample was to help ensure that studies were specifically related to the ongoing COVID-19 pandemic. Some of the studies assessed patient outcomes of routine care during the pandemic, although this was not a criterion for inclusion in the review.

### 2.2. Information Sources

The review team utilized four research databases to conduct the review: Cumulative Index to Nursing & Allied Health Literature (CINAHL) Complete, Scopus^®^, Complimentary Index and PubMed (which queries MEDLINE). Database searches were conducted between 1–5 November 2020.

### 2.3. Search

An initial Google and Google Scholar search was conducted by the researchers surrounding the overall topic surrounding the delivery of routine care during the COVID-19 global pandemic. The sample was limited to English-only and worked to identify main concepts and potential study variables to include in the research database postulated search string. While the Ebson B. Stephens Company (EBSCO host) search engine offers a programmed search suggestion (prepopulated) for COVID-19 and related terms, the Medical Subject Headings (MeSH) controlled vocabulary thesaurus for the National Library of Medicine did not offer related terminology for the “routine care”. Based upon the initial Google/Google Scholar search and multiple trial-and-error research database search queries by the research team, it was decided to simply use the term “routine” as a required search criterion in the review. This ensured a well-encompassed collection of potential articles in the study (yielding the highest initial article results). The final search string with Boolean operators utilized was: (“coronavirus” OR “COVID-19” OR “2019-ncov”) AND (“routine”).

### 2.4. Study Selection

Researchers met via a series of webinars to review identified articles and apply the review criteria specific to the research topic. A MS Excel spreadsheet hosted on an institutional cloud drive assisted in this collaboration process as initial search results were reviewed by the research team. The team worked to identify duplicate articles, books, other reviews, and publications that were not germane to the review topic. In the end, articles had to specifically focus on either a facilitator(s) and/or a barrier(s) to the delivery of routine care during the COVID-19 global pandemic to be included in the study sample.

### 2.5. Exclusion Criteria

**Figure 1:**
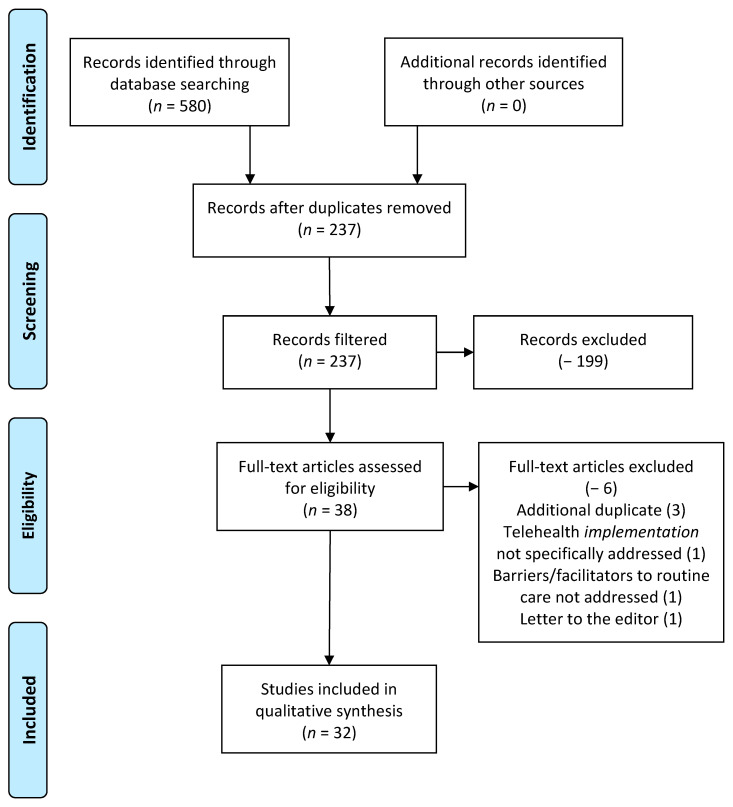
Preferred reporting items for systematic reviews and meta-analysis (PRISMA) figure that demonstrates the study selection process.

The study selection process with exclusion factors is illustrated in Figure 1. Upon completion of the screening process, at total of 32 articles were selected for inclusion in the review.

### 2.6. Risk of Bias

It is important to emphasize the broader scope of this review to identify initial facilitators and barriers to the delivery of routine care during the COVID-19 pandemic. Many of the articles identified met the criteria for this review, yet did not incorporate high levels of research method rigor (experimental design) and/or related findings. Instead, many articles focused upon an expert review of individual organization experiences and related processes regarding the delivery of routine care. As a result, the research team worked to evaluate, analyze, and classify identified facilitators and barriers related to the delivery of routine care, and potential patient outcomes (if available). Articles were selected for inclusion in this review independently by each researcher, and any discrepancies were resolved via discussion and with unanimous consent on the final sample.

## 3. Results

### 3.1. Study Characteristics

Each article in the sample was reviewed for participants involved, as well as identified facilitators and barriers experienced with the delivery of routine care during the pandemic. Additionally, specific patient outcomes related to routine care was also assessed, if included in the sample article. In the end, to be included in the review at least one facilitator and/or barrier had to be identified as the primary theme of the article. Provision of patient outcomes related to an article’s facilitator/barrier (or both) theme was not a required variable. Articles included in the review sample are listed in alphabetical order by author last name in Table 1.

The sample included both U.S. health care organizations, as well as international. A variety of research methods were utilized across the entire sample, specific participants were identified, as well as potential facilitators and barriers identified with the routine care during the ongoing pandemic. Additionally, any patient outcomes (if included) were also identified. If not addressed, “n/a” was inserted for that article in Table 1.

A summary of the underlying concepts surrounding the type and/or delivery of routine medical care identified in the review is shown in Table 2. Review of the 32 sample articles demonstrated fourth main types of routine health care services. Such underlying themes incorporate both facilitator and barrier variables, and also provide initial insight into the types of routine services affected by the pandemic, as identified in the literature. Several articles in the review possess more than one underlying facilitator and/or barrier related to its identified construct (not mutually exclusive to any one type of routine service).

The ‘Routine care, other’ overall construct identified in the literature summarizes routine care services similar to the initial construct (termed, ‘Chronic care management’), but excluding common chronic conditions such as heart disease, diabetes, and obesity. Examples of health services in this last category include conditions such as diagnostic imagining, preventive dental care, chronic (ongoing) orthopedic/arthritis care, and palliative care services.

### 3.2. Summary of Evidence

The pandemic has caused numerous limitations for healthcare employees to provide continuous, routine medical treatment for their patients. Research found notable impacts on rural and lower socioeconomic areas on a global scale [7]. Rural and lower socioeconomic areas prior to COVID-19 were limited in resources and healthcare options. Healthcare providers of all types noticed rapidly depleting resources, limiting their ability to provide care to their patients in routine care facilities [5]. Due to fear of possibly being infected with COVID-19, the prevalence of patient adherence to sustaining routine care encounters depleted substantially for organizations in the sample. However, other organizations identified the possibility of exposure to COVID-19 a calculated, assumed risk for other patients when it came to receiving routine care.

**Figure 2:**
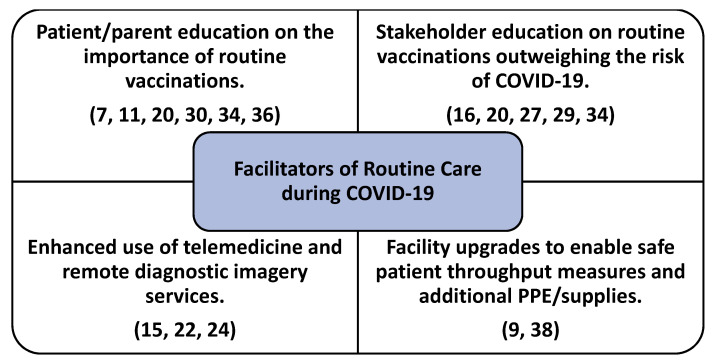
Underlying variables (constructs) identified as facilitators for the delivery of routine care during the pandemic.

**Figure 3:**
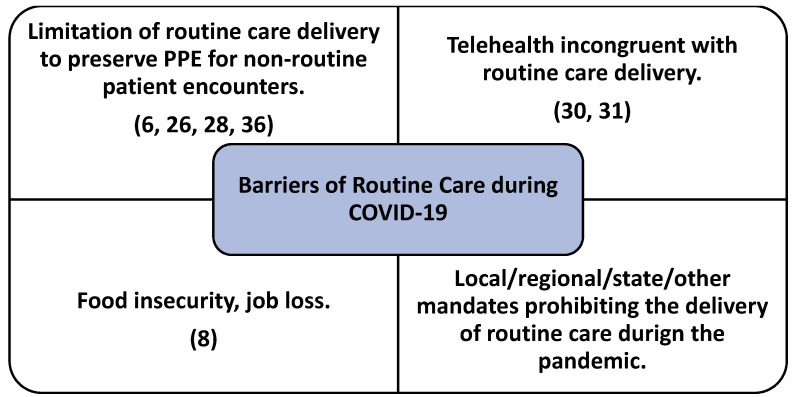
Underlying variables (constructs) identified as barriers for the delivery of routine care during the pandemic.

Figure 2 and Figure 3 demonstrate the underlying constructs identified in the sample regarding both facilitators and barriers to the delivery of routine care services during the global pandemic. In addition to the four construct categories identified for facilitator and barrier variables, the research team also discovered the importance of education for a variety of health care stakeholders (ex. patients, parents, and even providers) played a vital role in assisting with routine care delivery. Specifically, education of parents (or guardians of pediatric patients) was cited in the literature surrounding the importance of routine (non-COVID-19) vaccinations during the pandemic [11,20,30,36], while also playing an important role in demonstrating the necessity of routine vaccinations extending beyond the potential risk of contracting COVID-19 during routine vaccine administration processes [20,29,34].

## 4. Discussion

### 4.1. Facilitators of Routine Care

This review identified many facilitators to help further enable the provision of routine health care services. Constituting a majority of the overall routine care constructs identified in the literature (Table 1), the provision of routine (non-COVID) vaccinations was significantly increased using a multitude of factors. Patient education surrounding the importance of routine vaccinations outweighing risk of contracting COVID-19 was a primary finding in the literature to facilitate routine vaccinations [10,16,30,32]. Parental awareness (education) regarding the importance of routine vaccination, even during a global pandemic was also identified as a significant facilitator to delivery of care [10,32].

In addition to patient and other healthcare stakeholder education surrounding the facilitation of routine vaccinations, the use of telemedicine and remote diagnosis using medical imagery was also identified in the literature [15,22,24]. While some obstetrical care was directly related to diagnostic and similar imaging procedures, facilitation of this service line was also enhanced through the increased use of telehealth resources, as well as facility upgrades to enable safe patient throughput, adequate PPE, and additional medical supplies [9,38].

### 4.2. Barriers to Routine Care

Health service categories recognized by the research team in Table 1 were also analyzed for potential barriers to routine care as preempted by COVID-19. When the pandemic began just over a year ago, adequate and appropriate PPE was of concern and challenged the healthcare industry significantly. Such constraints limited the amount of care physicians could safely provide. These initial limitations consequently placed pressure on who care should be prioritized for, including delivery of routine care services [6,26,28,36]. While PPE was cited as a routine care delivery constraint (barrier) in a majority of the articles in the review, the specific reference to the limiting of routine (including elective) care in an attempt to preserve PPE for non-routine, acute-care encounters was identified as a significant routine care barrier.

The increased use of telehealth during the global pandemic has assisted in the facilitation of health care delivery, to include routine care [39]. However, as specialty-specific clinical practice guidelines are continuing to develop and be implemented by providers of all industry segments, use of telehealth is not a 100% comprehensive solution to facilitate the provision of routine care. Identified as an inappropriate method of care delivery for specific types of routine health care services, and/or not accepted as a preferred method of treatment, telehealth was classified as a barrier to routine care in some routine treatment circumstances [30,31].

An important, non-clinical, yet key variable identified in the review surrounds those seeking routine health care services during the global pandemic who are also encountering significant socioeconomic challenges. While only identified in one article in the review [8], the practical significance food insecurity during the COVID-19 global pandemic is considered highly influential in a person’s decision to delay their routine health care service appointments [8]. Food pantry and other assistance measures continue to try and alleviate the pressures put on families during this difficult time, placing the scheduling and follow-on procurement of routine health care services much lower on the priority list of daily tasks for many [40]. An ongoing challenge in the United States and beyond [40], often the delayed routine care observation, because of food insecurity, is most-often also correlated with the person’s employment status (i.e., job loss) [8].

Another barrier to the delivery of routine care is local/regional/state/other public health mandate or governmental policy prohibiting the delivery of routine care during specific shut-down stages of the pandemic. Here, while understood as a barrier to the delivery of routine care and important to note in this review, it is not a process variable (care obstacle) that is able to be successfully managed by the healthcare organization, succumbed to appropriately following policy guidelines and related public health precautions.

### 4.3. Expansion of Telehealth: Pros and Cons

Use of telehealth services has become an essential care delivery method during the pandemic for both routine and acute care services [39]. While enhancing telehealth capabilities for the delivery of routine care has been identified as a care delivery facilitator by the research team (Figure 2), specific types of primary (routine) health care services have been identified in the literature as inappropriate when delivered solely using telehealth services (Figure 3). Healthcare organizations are urged to research their specific routine care delivery patient needs and determine the suitability of telehealth use in lieu of in-person visits. Regardless, the observed expansion of telehealth services offers both pros and cons as observed in the literature, even beyond the scope of this study specifically focusing on routine care delivery.

Offering a safe treatment modality (accommodating physical distancing), many patients comment on the experienced safety and provider consideration for COVID-19 precautions when using telehealth services [41]. Patient (and provider) convenience are also benefits identified in the literature [42,43]. Additional conveniences include elimination of some opportunity costs encountered with in-person visits, as compared to virtual visits (ex. missing work, travel expenses, etc.).

Alternatively, the enhanced use of virtual visits has resulted in some disadvantages as experienced by healthcare stakeholders. While many healthcare privacy requirements have been adjusted in an attempt to adapt to the global pandemic and enforce physical distancing, the concept of informed consent, patient privacy, and patient confidentiality are still of concern [42,44,45]. The expansion of telehealth has even been identified as a contributor to enhanced social isolation for specific patient population segments who prefer in-person (non-virtual) collaboration with their medical providers [41].

### 4.4. Limitations

As with any research study, this review’s findings also possess limitations. The researchers only investigated articles with publication dates within the 1 January 2020 through 31 October 2020 timeframe. While the intent was to limit the identified sample to only COVID-related publications, this did significantly limit the initial search findings. Only articles published in peer-reviewed journals were included in the study, and various study methods and associated levels of rigor related to the investigation of routine care delivery varied significantly across the final review sample. Reviewing articles only available in English also limited the sample. As the healthcare industry continues to work improving access and quality of care related to routine service delivery, additional investigation in this topic is necessary and a strong opportunity for future research.

## 5. Conclusions

The provision of routine health care services during the COVID-19 global pandemic is challenged by barriers that can be overcome with planning and community effort [7,10,16,29]. Identified routine care delivery facilitators demonstrate the healthcare industry’s ability to adapt to public health physical distancing and other COVID-19 precaution measures while still providing adequate care for the routine patient [11]. While many healthcare organizations have been successful with their attempt to adjust delivery of routine care processes to establish a continuity of care, an important organizational leadership decision must also be made regarding when to halt routine care delivery to focus on acute COVID-19 patients when limitations exist [35].

This research provides an initial investigation into facilitators and barriers related to the provision of routine care during COVID-19. Identified themes can assist healthcare organizations with future planning to address such concerns, while specific routine care delivery processes and procedures will be dependent upon organization type and other inherent organizational characteristics. Management of chronic conditions [13,23,25,35], obstetrical care [9,31] and other routine care [14,18,21,26] have been identified as successful routine care delivery services continued during COVID-19. Still, a recognized backlog of delayed routine care will require additional industry initiatives and adaptations as vaccinations continue to be administered and the pandemic eventually subsides.

## Figures and Tables

**Table 1:** Summary of Findings (*n* = 32).

Author(s)	Study Participant(s)	Facilitators to Routine Care	Barriers to Routine Care	Patient Outcome(s)
Abbas et al. [7]	Pediatric patients in Africa seeking routine immunizations during the pandemic	Health benefits (prevention of estimated deaths) outweighed the risk of COVID-19 spread for clinic visits.	Lack of COVID-19 vaccine and other available immunizations resulted in an inability to perform routine care and increase the estimated mortality rate.	Sustainment of routine vaccinations and prevention of related diseases outweigh COVID-19 exposure risk.
Abrams et al. [8]	Families with children experiencing food insecurities at Federally Qualified Health Centers (FQHCs) during the pandemic	n/a	Food insecurity experienced by 60% of survey respondents. Fear of running out of food experienced by 90% of respondents.	Food insecurity affected patients’ ability to receive routine care during the pandemic at FQHCs.
Abu-Rustum et al. [9]	Obstetric patients	A tiered system was created to prioritize pregnant mothers needing obstetric examinations to ensure ongoing access to care.	Special modifications and a tiered system left some mothers prioritized at a lower level, thus delaying care.	General guidance issued by the ISUOG organization to ensure the highest acuity patients were seen first in a controlled environment to prevent the spread of COVID for each possible patient circumstance.
Adamua et al. [10]	Pediatric patients in Africa	Systems thinking an analysis demonstrates the importance of ongoing routine vaccinations in Africa, outweighing the risk of COVID-19 in the country. System re-design was implemented.	A lack of analysis on the CLD (causal loop diagram) in the study suggested potential COVID-19 spread at local clinics for routine vaccinations.	Improved control over COVID-19 outbreaks by analysis of communities, population groups, and socioeconomic data.
Alsuhaibani and Alaqeel [11]	Pediatrics patients in Saudi Arabia	Improved parental awareness regarding the importance of routine vaccinations led to a decreased in the prevalence of delayed routine childhood immunizations.	Lack of parental education on the importance of routine childhood immunizations and delayed appointments due to COVID-19 precautions.	While most parents had a positive perspective on routine vaccinations, 65% of parents were not concerned about ongoing delays for routine care (vaccinations).
Abbasian et al. [12]	COVID patients (and non-COVID patients) requiring CT imaging procedure(s)	Computerized automated detection (CAD) applications assist in the speed of diagnosis of COVID infections amongst other atypical/viral pneumonia diseases.	Risk of the spread of COVID-19 during the CT procedure was of concern.	Faster diagnosis of patients with lung conditions related to COVID-19.
Berger-Richardson and Hong [13]	Patients with breast cancer	Treatment recommendations for patients with breast cancer enabled a safe environment of care to help prevent the spread of COVID-19.	A delay in endocrine treatment may lead to the risk of progression in these patients.	Delays to surgical treatments for routine cancer care longer than 3–6 months require ongoing monitoring and imaging as precautional measures.
Carmelo et al. [14]	Patients requiring radiology/imaging during routine dental care procedures	Remote diagnosis using medical imagery is recommended versus in-person to avoid the spread of COVID-19. Additional procedures (scheduling, use of additional PPE, etc.) also was implemented.	Dentistry is prone to a high risk of transmissibility when precautions and remove diagnoses are not conducted.	Patient, as well as clinical staff and providers were able to help control the risk of spreading COVID-19.
Chan et al. [15]	Patients requiring a head and neck exam during the pandemic	Specific treatment guidelines established in order to treat patients requiring head and neck procedures.	Nonessential head and neck patients were categorized, and care postponed during the pandemic.	Lower-level patients (non-urgent, low acuity) experience an access to care issue during the pandemic.
Chandir et al. [16]	Patients requiring routine immunizations in Pakistan	A quantitative analysis (pre- and post-study) reveals that returning to normal social and school events will help facilitate routine immunization rates in rural/poorer regions.	Global lockdown due to the pandemic as related to high/low-income countries led to lower routine vaccination rates.	n/a
Chudasma et al. [17]	Healthcare providers (global survey)	New methods of delivering care in a virtual environment is necessary for the ongoing routine care related to chronic diseases.	A reduction of in-person visits due to the pandemic initially led to a decrease in the treatment of routine, chronic conditions.	Patients with chronic conditions able to receive care in the virtual environment, versus not receiving the required routine care at all.
Corden et al. [18]	Patients with routine dermatology referrals	Patient care was deemed adequate during temporary process changes through the use of patient triage and indication of COVID-19 diagnosis.	Inefficiencies noted regarding the temporary protocols established to care for these patients.	Adequate patient care established for these industry segment, while temporary process changes identified the need for follow-on, long-term solutions as the pandemic continues.
Desai et al. [19]	Patients requiring electrodiagnostic testing	Staying current on CDC compliant measures is required to ensure provider safety. Further organizational committee work (collaboration) to establish protocols helped ensure preventive measures were followed.	n/a	Patients requiring this routine diagnostic test were able to receive it.
Dinleyici et al. [20]	Pediatric patients requiring routine vaccinations during the pandemic	Analysis of COVID-19 mitigation and routine immunization services allows for an assessment of already high-risk children during the pandemic.	Children with diseases considered ‘under control’ prior to the pandemic will be put at higher risk if the population slows routine vaccinations.	n/a
Ekbert et al. [21]	Pediatric patients receiving palliative care	An assessment of communication between providers and patients requiring routine palliative included the relevance of COVID-19 in the treatment process.	Clinical disposition due to COVID-19 was identified at 55% and parents identified at 45%.	COVID-19 was identified as a persuasive treatment variable during provider communications with routine palliative care patients.
Antonio et al. [22]	Patients requiring routine endoscopic procedures	Additional PPE, separation of procedure rooms, and methods regarding room preparation assist in the return of routine endoscopic procedures during the pandemic.	Lack of quality control and detailed measures to follow will lead to increased transmissions during routine care.	n/a
Fung et al. [23]	Pediatric patients requiring routine diabetes care	Use of telephone only and also virtual (webinar) patient-provider visits enable access to routine care.	n/a	Survey results demonstrated patient and family member satisfaction with both telephones only and online/webinar (virtual) patient visit methods.
Gupta et al. [24]	Patients requiring routine endoscopic procedures	Routine point-of-care testing for COVID-19 and clinical risk assessments assisted with access to this procedure.	Patients requiring an elective endoscopic procedure were prioritized lower and/or postponed.	Access to care for high-acuity patients, while elective patients were recommended to be postponed due to the risk assessments.
Howley et al. [25]	Patient with Lemierre syndrome and delayed access to routine care	Enhanced awareness to the provider community regarding how delayed access to routine (‘non-essential’) care can lead to a complex illness otherwise treatable.	Delayed routine care resulted in a complex medical condition.	Patient’s disease/acuity progressed due to delayed routine care.
Jung et al. [26]	Medical providers and their use of PPE when providing routine care to COVID-patients	Adherence to proper protocols regarding the dawning and removal of PPE will help lower infection rate of medical providers.	PPE contamination was identified on the top of provider heads, dorsum of the foot, wrist, and abdomen.	Identification of medical provider PPE dawning/removal of PPE protocols assists with reducing COVID-19 contamination.
Kinoshita and Tanaka [27]	Infants receiving the Bacille Calmette-Guérin (BCG) vaccination	Awareness efforts encouraged by health officials assist in keeping the high level of vaccinations during the pandemic.	Challenges for BCG vaccination presented due to COVID-19, distancing, and vaccination processes.	Spread of tuberculosis was lowered after attention and re-education of how important routine vaccinations are during the pandemic.
Landgon-Embry et al. [28]	New York City routine childhood vaccination rates	Increased awareness efforts led to better routine vaccination compliance during the severe outbreak in the city.	Significant COVID-19 spread and also patient/family fear of routine care lowered routine vaccination rates across the city.	Increased education and distancing/safety protocols increased routine vaccination compliance in the city.
MacDonald et al. [29]	Canadian patients requiring routine vaccinations	Specific follow-up (patient communications/contact methods) for those who are due or missed a routine vaccination increase the compliance with this program.	Preventable diseases threatened to increase in prevalence if routine vaccinations are not continued during the pandemic.	Contacted patients educated on the importance of routine vaccination compliance and safety protocols in-place during COVID-19.
McDonald et al. [30]	Children in England	Parental education on routine vaccinations helped to increase compliance during the pandemic around week 17.	Physical distancing requirements led to delayed and canceled routine vaccination appointments as identified by the organization’s EMR system.	Physical distancing messaging and related communications can dramatically affect the compliance with routine vaccinations/care.
Meyer et al. [31]	Patients requiring routine obstetrical care in Israel	Analysis of routine obstetrical care referrals, discharge rate, time spend in an ED/ER, delivery unit and/or admission rate revealed information to assist in identifying and correcting obstetrical patients missing routine care appointments.	Due to physical distancing requirements, routine appointments for obstetrical care were avoided by female patients and an increase in ER/ED deliveries was also experienced.	Avoided routine obstetrical care led to an increase in ER/ED deliveries in Israel during the pandemic.
Ogundele et al. [32]	Patients requiring routine vaccinations in Nigeria	Observations regarding the Nigerian health system document a need to prioritize routine childhood immunizations to help alleviate already-low vaccination rates (pre-pandemic).	Communities still struggle with compliance with routine care and COVID-19 exacerbated the issue.	Hinderance on facilitating vaccinations in Nigeria will lead to an increase in morbidity and mortality due to a lack of routine care.
Papanstasiou [33]	Nuclear medicine providers/staff	A review of facility upgrades (additional hand sanitizer stations, plastic screens, additional PPE to be handed-out, and ensuring sufficient radio pharmaceutical supplies) helped to re-established delivery of care.	Lack of intervention and initiatives would have led to a shortage of nuclear medicine routine care.	Patients requiring routine nuclear medicine care were able to be seen as a result of these protocol changes.
Ranganthan and Khan [34]	Children in India	A recommendation of future telecommunication initiatives, as well as community workers and use of social media may help increase future routine vaccination compliance in challenging times.	A hesitation to give routine vaccinations in India by parents and providers initially led to a decrease in compliance.	Use of government messaging/communications enhanced routine vaccination rates in India after issues related to COVID-19.
Rimmer [35]	General practitioners	A recommendation of delaying routine care for patients over 75 led to additional time/availability for these providers to assist with COVID-19 patients.	Delayed care for those over 75 without a COVID-19 diagnosis was an implication of the recommendation.	General practitioner availability to assist with COVID-19 patients increased.
Saxena et al. [36]	Patients requiring routine vaccinations	Compliance with getting routine vaccinations on-time will ensure their efficacy.	n/a	If a shot is delayed, then it may no longer be effective as a vaccine for the patient.
Seyahi et al. [37]	Patients seeking routine care for rheumatic diseases in Turkey	The mental state of patients with rheumatic diseases receiving and/or canceling routine care during the pandemic was better than expected.	n/a	Patient routines changes, to including some stopping rheumatic medications altogether during the pandemic.
Zangmo et al. [38]	Obstetric patients requiring antenatal care	Increased use of telemedicine assisted in the continuation of this care to avoid face-to-face visits without sacrificing quality of care.	COVID-19 restrictions can decrease communication between the patient and provider.	Methods were established to help identify better methods of care for obstetric patients.

**Table 2:** Summary of Overall Health Care Service Constructs Identified (*n* = 32).

Identified Health Care Service Constructs in the Sample	Accurrence of Attribute (%)	Frequency of Occurrance (%)
Chronic care management (health disease, diabetes, obseity)	12, 13, 16, 17, 23, 25, 35, 37	8 (25%)
Obstretical care	9, 31, 38	3 (9%)
Routine (non-COVID-19) vaccinations	7, 10, 11, 16, 20, 27, 28, 29, 30, 32, 34, 36	12 (37%)
Routine care, other	8, 14, 15, 18, 19, 21, 22, 24, 26, 33	10 (31%)
